# Changes in mean scrotal circumference in performance tested Swedish beef bulls over time

**DOI:** 10.1186/1751-0147-54-74

**Published:** 2012-12-15

**Authors:** Pernilla Eriksson, Nils Lundeheim, Lennart Söderquist

**Affiliations:** 1 Department of Clinical Sciences, Division of Reproduction, Swedish University of Agricultural Sciences (SLU), Box 7054, Uppsala, SE-750 07, Sweden; 2 Department of Animal Breeding and Genetics, Swedish University of Agricultural Sciences (SLU), Box 7023, Uppsala, SE-750 07, Sweden

**Keywords:** Andrology, Cattle, Genetics, Testicles, Scrotal circumference, Beef bull

## Abstract

**Background:**

There is a growing interest in beef cattle breeding in Sweden. The majority of the females are bred naturally, which is why it is important to choose healthy fertile bulls to obtain good reproduction and profitability. The breeding soundness evaluation includes measurement of scrotal circumference (SC). Our aim was to analyze if the SC of performance tested beef bulls has changed over the years. In total, 1332 bulls (Angus, Charolais, Hereford and Simmental) from 13 batches (1997-2010) were included in the study. Case book entries from final evaluation of the bulls, 11-13 months old, were compiled and analyzed.

**Results:**

An overall mean SC of 34.7 cm independent of breed and age was found which is above the set minimum level. Only eleven bulls did not reach the minimum level. An increase in SC of 0.06-0.07 cm/year was shown for all breeds. In all (1997-2010), the increase of the average SC (independent of breed and age) was approximately 1 cm. The positive trend was apparent for all breeds but only statistically significant for the Charolais breed.

**Conclusions:**

In conclusion, our results indicated an increase in the SC over time, which improves the possibilities to obtain performance tested beef sires in Sweden with the potential for achieving better fertility results.

## Background

Beef production is currently an increasing agricultural sector in Sweden with more than one third (192 501) of all Swedish cattle being beef breeds [1]. The majority of the females (> 98%) are bred naturally [2] with the intention of obtaining one live calf per female and year. Thus, selection of herd sires is a critical decision affecting reproductive performance and profitability, since the fertility of the bull is of greater importance for the total pregnancy result than that of the individual female [3]. Sub-fertile bulls delay conception, prolong the calving season, reduce calf weaning weights and increase female culls [4]. It is thus important to examine the bull’s potential fertility, by applying a so called *Bull Breeding Soundness Evaluation (BBSE)* procedure, where a general health check, an andrological evaluation, collection of a semen sample and an evaluation of the bull’s mating ability are included [3]. In the andrological evaluation, the scrotal circumference (SC), among other parameters, is measured. Several studies (e.g. [5-7] have shown a high correlation between scrotal circumference and paired testes weight, which is correlated with daily sperm production and semen quality, thus making the scrotal circumference an indirect measurement of the sperm-producing capacity of the testicles. Bulls with large SC have half-sib heifers and daughters with earlier puberty and greater fertility (reviewed by [8]). Several studies have shown a moderate correlation between breed means for yearling SC and age at puberty in heifers and a high correlation to the fertility of female offspring, which is why SC responds well to selection (reviewed by [8]). Unfortunately, the majority of the Swedish beef bulls used for breeding are still bought without first having undergone a BBSE.

Previously, (until 2010/11), approximately 170 beef bull calves were tested annually at the performance station in Gismestad near Linköping, Sweden. The bull calves were between five to eight months old at arrival and the testing period was approximately six months. They were selected by each breeding organization based on their BLUP (Best Linear Unbiased Prediction)-values. At the performance testing station the bull calves were gathered in different groups according to breed and were raised together under the same conditions, being evaluated for growth rate, health, sustainability and temperament. Since traditionally there was a focus on their growth rate, the bulls were weighed every second week. In connection with weighing, the testes and epididymides were palpated. If defects or deviations were found at the examination, the bulls were culled. Approximately six months later, when the bulls were between 11 and 13 months old, when only approximately half of the initial number of bulls remained, a final measurement of the scrotal circumference (SC) was done to see if they reached the minimum threshold values set for SC for each breed, and the genital organs were examined for the last time just before the yearly auction (Lilja-Ambuhm M, personal communication, 2010). Since the bulls were sold at the auction at a relatively low age (11-13 months) almost half of them were considered not to have a completely normal semen picture yet [9], and since no semen samples were collected at the simplified BBSE applied at the performance testing station, the palpation findings and the scrotal circumference measurements were the only indicators of the bulls’ potential fertility. It has, however, been shown that bulls with a small SC at one year of age did not catch up over time and would still have small SC measurements at 2 years of age. Therefore, final decisions based on SC could be made by the time bulls are approximately 12 months old [10].

The aim of this study was to analyze the change, and possible trend, in the mean scrotal circumference of batches of Swedish performance tested beef bulls at around 365 days of age over time (1997-2010).

## Methods

In total, 1332 bulls from four of the most common beef breeds in Sweden (Angus, n = 120; Charolais, n = 713; Hereford, n = 216 and Simmental, n = 283) from 13 batches of bulls from 1997 to 2010 were included in the study. All bulls included were housed at the same performance testing station (the only one in Sweden) and on arrival, at approximately six months of age, they were divided into groups of 10-20 animals, based on breed and body weight. Each group was kept in a semi-outdoor pen with a concrete floor and straw as bedding. They were fed a complete-ration feed *ad libitum* with approximately 60% of the energy provided by silage and 40% by concentrate. The bulls were weighed every second week throughout the testing period (September-March) and at the end of the period, an individual growth index was calculated. Besides growth rate, conformation, temperament and SC were evaluated. The minimum threshold values for SC at 365 days of age used at the performance testing station were for Angus/Charolais 31 cm, Hereford 30 cm and for Simmental 29 cm. Case book entries from the final evaluation occasion of the bulls, before the auction, were compiled and analyzed. The clinical evaluations and scrotal circumference measurements were all made by the same person during the whole study period (1997-2010). First the gait of the bull was assessed at a walking pace and at trot and then the bull was placed in a crush and the testes and epididymides were carefully palpated, the size of the testes assessed, the prepuce inspected and the scrotal circumference measured with a metal scrotal tape (Sullivan Supply Inc; Dunlap, Iowa, USA).

### Statistical analyses

Data were handled and analyzed using the SAS-program (ver. 9.2, SAS Inst. Inc., Cary, NC, USA). Mean values, standard deviation (SD) and correlations were calculated, and the variation in scrotal circumference was analyzed using analyses of variance (PROC GLM) according to a statistical model including the effects of breed, and the linear regressions within breed of age, year and weight at evaluation. P-values < 0.05 were considered to be significant.

## Results

**Figure 1:**
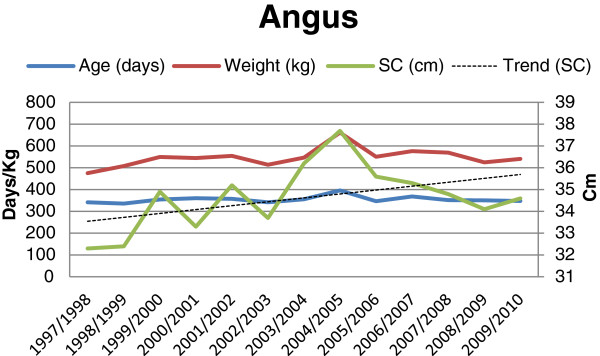
Age (days), weight (kg) and scrotal circumference (cm), and trend for change in scrotal circumference for Angus bulls (n = 120) at final examination during 1997-2010.

**Figure 2:**
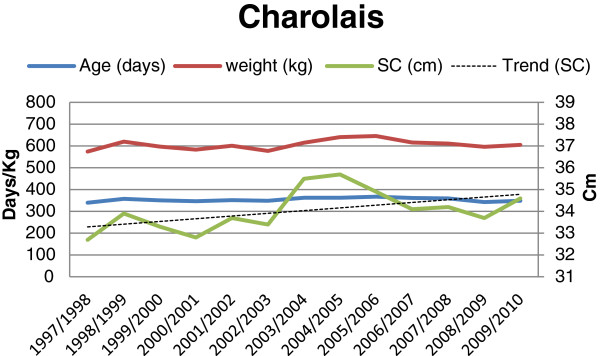
Age (days), weight (kg) and scrotal circumference (cm), and trend for change in scrotal circumference for Charolais bulls (n = 713) at final examination during 1997-2010.

**Figure 3:**
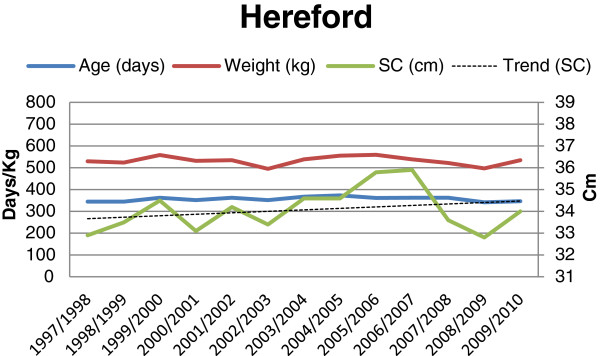
Age (days), weight (kg) and scrotal circumference (cm), and trend for change in scrotal circumference for Hereford bulls (n = 216) at final examination during 1997-2010.

**Figure 4:**
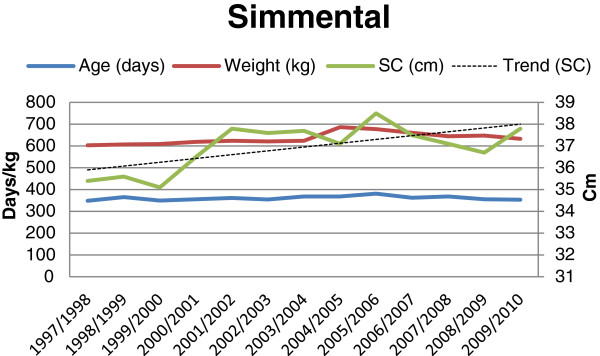
Age (days), weight (kg), scrotal circumference (cm), and trend for change in scrotal circumference, for Simmental bulls (n = 283) at final examination during 1997-2010.

The results showed an overall mean scrotal circumference of 34.7 cm. The mean SC (± SD) for the different breeds and batches are shown in Table 1. The overall mean (± SD) age and weight of the bulls at the time of the final evaluations were 356 days (± 21.0) and 595.6 kg (± 61.8), respectively. Average age, weight and mean scrotal circumference for each breed and year are shown in Figures 1, 2, 3 and 4. The average (± SD) scrotal circumference adjusted to 365 days (according to [11]) for the different breeds during 1997-2010 were 36.6 cm (± 2.2) for Hereford, 34.8 cm (± 2.3) for Charolais, 35.4 cm (± 2.2) for Angus and 37.4 cm (± 2.4) for Simmental.

**Table 1:** Average (± SD) for scrotal circumference (cm) for the different breeds and years studied (n = 1332)

***Batch***	**Angus n**		**Charolais n**		**Hereford n**		**Simmental n**		**Bulls (n), total**
*1997/1998*	32.3 ± 1.8	7	32.7 ± 2.0	58	32.9 ± 2.7	20	35.4 ± 1.7	16	101
*1998/1999*	32.4 ± 0.5	5	33.9 ± 2.4	47	33.5 ± 2.3	23	35.6 ± 2.0	13	88
*1999/2000*	34.9 ± 1.5	11	33.3 ± 1.9	41	34.5 ± 2.3	19	35.1 ± 2.5	14	85
*2000/2001*	33.3 ± 2.1	10	32.8 ± 2.7	43	33.1 ± 2.2	16	36.5 ± 1.9	14	83
*2001/2002*	35.2 ± 3.2	11	33.7 ± 2.4	70	34.2 ± 1.5	16	37.8 ± 2.6	33	103
*2002/2003*	33.7 ± 3.1	7	33.4 ± 2.3	37	33.4 ± 2.3	9	37.6 ± 3.1	12	65
*2003/2004*	36.2 ± 2.2	13	35.5 ± 2.6	70	34.6 ± 2.3	12	37.7 ± 2.4	31	126
*2004/2005*	37.7 ± 2.1	3	35.7 ± 2.3	42	34.6 ± 2.3	10	37.1 ± 2.8	16	71
*2005/2006*	35.6 ± 2.1	10	34.9 ± 2.3	57	35.8 ± 2.4	10	38.5 ± 2.0	21	98
*2006/2007*	35.3 ± 1.9	10	34.1 ± 2.9	56	35.9 ± 2.4	18	37.5 ± 3.5	26	110
*2007/2008*	34.8 ± 2.3	9	34.2 ± 2.2	62	33.6 ± 2.3	21	37.1 ± 2.7	30	122
*2008/2009*	34.1 ± 1.4	13	33.7 ± 2.4	61	32.8 ± 2.1	20	36.7 ± 2.0	28	122
*2009/2010*	34.6 ± 2.6	11	34.6 ± 2.4	69	34.0 ± 2.1	22	37.8 ± 1.9	29	131
***In total***	**34.7 ± 2.4**	**120**	**34.1 ± 2.5**	**713**	**34.0 ± 2.4**	**216**	**37.2 ± 2.6**	**283**	**1332**

Positive correlations were found across breeds between scrotal circumference and age (r = 0.42, p < 0.0001), between scrotal circumference and weight (r = 0.49, p < 0.0001) and between weight and age (r = 0.55, p < 0.0001).

For the Charolais breed a statistically significant increase in the scrotal circumference was seen during the studied period (0.073 cm/year, p < 0.0008) after adjusting for age and weight. For the other breeds (Angus, Hereford and Simmental, respectively) the same trends were seen, in absolute figures, (0.063 cm/year, 0.065 cm/year, and 0.064 cm/year, respectively), but the increase was not statistically significant (p < 0.247, p < 0.074, and p < 0.085, respectively).

In total, 11 out of 1332 bulls did not reach the minimum levels for scrotal circumference stipulated at the performance testing station at the time of the final evaluation during 1997-2010.

## Discussion

The average scrotal circumferences for the performance tested bulls were 34.7 cm independent of breed and age. The average scrotal circumference, adjusted to 365 days of age for the different breeds, was always above the minimum SC threshold values set for approval at the performance testing station. The averages of the scrotal circumference in our study were, in general, higher than those in earlier studies, complied by Barth [11]. One reason for this might be that the minimum threshold values used at the performance testing station in Sweden are higher than the Canadian recommendations for minimum scrotal circumference values, which probably have led to a more pronounced selection towards larger scrotal circumferences in our study. However, the majority of the studies on which Barth (2000) based his compilation were performed between 20 to 40 years ago; hence the SC measurements of those studies might not be considered completely relevant in comparison with scrotal circumference measurements found today. In addition, Barth [11] emphasizes that ultimately bulls should be selected for a scrotal circumference that is in line with the average or higher, instead of just reaching the set minimum SC threshold values (usually based on the mean minus 2 standard deviations), in the same way as selections for growth rate and other traits are made, to hasten breeding progress.

Only a few bulls, 11 out of 1332 during the studied 13 batches of bulls, were culled at the final evaluation due to too low scrotal circumference. However, the bulls that were culled earlier due to too low scrotal circumference before the final evaluation are not included, which makes the total number of rejected bulls somewhat higher. Since it only concerns very few bulls altogether during the whole time period, according to the veterinarian in charge (Lilja-Ambuhm M, personal communication, 2010), the total number of bulls that did not fulfill the minimum threshold values set for the SC at the station was still very low.

Our findings concur with previous observations that an increased age results in a higher body weight and also to some extent in a higher scrotal circumference [12], since a certain correlation can be seen among these parameters. Apart from age and weight, other factors can also influence the scrotal circumference measurements of the bulls in the different batches, such as ambient temperature at the time of examination [13]. Throughout the years, the final evaluation has taken place in February and March in the semi-outdoor barn at Gismestad. Recordings of the prevailing temperatures in the barn at the time for the final evaluation are, unfortunately, not available and therefore the influence of a possible suboptimal ambient temperature cannot be assessed in our study.

The results from our study showed, however, that there has been a gradual increase in the scrotal circumference of 0.06-0.07 cm/year for all breeds during the studied period (1997-2010). In total, the increase in the average scrotal circumference (independent of breed and age) among young Swedish performance tested beef bulls was almost 1 cm during these 13 years. Although the positive trends over time were similar for all studied breeds in absolute figures, the increase was only statistically significant for the Charolais breed, which was numerically over-represented compared to the Hereford, Angus and Simmental breeds.

Barth (personal communication, 2010) recently made a similar investigation regarding performance tested Angus-bulls in Canada and concluded that there had been an increase in the SC of approximately 1 cm during the last 15 years, which is accordance with our results. This indicates that despite using only the set minimum SC threshold values for selection purposes, a certain increase in the scrotal circumference can be reached over time. In Canada, however, some breeders have taken into account the high heritability of scrotal circumference, and used the average values for the breed in question when making their sire selections instead of the minimum scrotal circumference values, thereby achieving considerable breeding progress by their strategy (Barth 2000).

In many countries electro ejaculation is routinely used when conducting BBSE but this technique is prohibited on unanaesthetized animals in Sweden and in many other European countries [14]. Collection of semen using an artificial vagina is by far the most common method used in Swedish dairy bulls. However, this method is considered too difficult, time consuming and sometimes dangerous for routine semen collection from untrained beef bulls under field conditions. Consequently, semen from yearling beef sires is not evaluated at all before the bulls are offered for breeding purposes. Breeding for a larger SC ought, in the long run, to result in a larger proportion of bulls having reached puberty and a normal sperm quality at the time for their final evaluation at about 365 days of age, than the 48% that Persson and co-workers reported earlier [9] based on assessments of semen samples obtained from post mortem examination of genital organs from bulls at the performance testing station during 1999-2003. A larger proportion of sexually mature bulls at the time of the final evaluation at approximately 12 months of age would facilitate the application of a complete BBSE procedure, whereby a semen sample collected by transrectal massage could be routinely included [15]. It is generally accepted that sires with above-average SC produce female offspring that reach puberty earlier (0.75-10 days) and have greater lifetime reproductive potential. A possibility to select for bulls with larger testicles could be to start to use a certain scrotal circumference value for each bull relative to all the other bulls at the performance testing station within the same breed and batch in line with the value that is currently calculated for the growth rate of the individual bull.

Our study showed that the average scrotal circumference measurements for the performance tested beef bulls in Sweden were somewhat higher than those internationally set and that very few bulls did not reach the minimal SC requirements. However, the vast majority of beef bulls used for breeding purposes has not been subjected to any BBSE at all. Thus, information about scrotal circumference measurements is lacking for the majority of the beef bulls used for breeding in Sweden today. This is unfortunate and needs to be more carefully addressed by the breeding organizations in Sweden and also by the insurance companies that, at present, apply no minimum SC threshold values before insuring beef bulls.

## Conclusions

Our results indicate that it is possible to increase the SC in Swedish performance tested beef bulls over time by applying a minimum threshold value for the scrotal circumference. This trend for an increase in the SC over time may have also improved the possibilities to obtain performance tested beef sires at the auction with increased potential for achieving better fertility results.

## Competing interests

The authors declare that they have neither financial nor non-financial competing interests.

## Authors' contribution

PE participated in the design of the study, collected and analyzed the data, and drafted the manuscript. NL performed the statistical analysis and helped to draft the manuscript. LS participated in the design and coordination of the study, analyzed the data and helped to draft the manuscript. All authors read and approved the final manuscript.
